# The Neural Basis of Major Depressive Disorder in Adults: A Meta-Analysis of Functional Magnetic Resonance Imaging Activation Studies

**DOI:** 10.1192/j.eurpsy.2023.388

**Published:** 2023-07-19

**Authors:** A. M. Klassen, C. Baten, J. H. Shepherd, G. Zamora, S. Saravia, E. Pritchard, Z. Ali, J. Jordan, S. K. Kahlon, G. Maly, M. Duran, S. L. Santos, A. F. Nimarko, D. W. Hedges, J. P. Hamilton, I. H. Gotlib, M. D. Sacchet, C. H. Miller

**Affiliations:** ^1^Psychology, California State University, Fresno; ^2^Psychology, Stanford University, Stanford; ^3^Psychology, Brigham Young University, Provo, United States; ^4^Biomedical and Clinical Sciences, Linköping University, Linköping, Sweden; ^5^Meditation Research Program, Psychiatry, Massachusetts General Hospital, Harvard Medical School, Boston, United States

## Abstract

**Introduction:**

Major depressive disorder (MDD) is a highly prevalent mental illness that often first occurs or persists into adulthood and is considered the leading cause of disability and disease burden worldwide. Unfortunately, individuals diagnosed with MDD who seek treatment often experience limited symptom relief and may not achieve long-term remission, which is due in part to our limited understanding of its underlying pathophysiology. Many studies that use task-based functional magnetic resonance imaging (fMRI) have found abnormal activation in brain regions in adults diagnosed with MDD, but those findings are often inconsistent; in addition, previous meta-analyses that quantitatively integrate this large body literature have found conflicting results.

**Objectives:**

This meta-analysis aims to advance our understanding of the neural basis of MDD in adults, as measured by fMRI activation studies, and address inconsistencies and discrepancies in the empirical literature.

**Methods:**

We employed multilevel kernel density analysis (MKDA) with ensemble thresholding, a well-established method for voxel-wise, whole-brain meta-analyses, to conduct a quantitative comparison of all relevant primary fMRI activation studies of adult patients with MDD compared to age-matched healthy controls.

**Results:**

We found that adults with MDD exhibited a reliable pattern of statistically significant (p<0.05; FWE-corrected) hyperactivation and hypoactivation in several brain regions compared to age-matched healthy controls across a variety of experimental tasks.

**Conclusions:**

This study supports previous findings that there is reliable neural basis of MDD that can be detected across heterogenous fMRI studies. These results can be used to inform development of promising treatments for MDD, including protocols for personalized interventions. They also provide the opportunity for additional studies to examine the specificity of these effects among various populations-of-interest, including youth vs. adults with depression as well as other related mood and anxiety disorders.

**Disclosure of Interest:**

None Declared

